# *Retracted:* Kale (*Brassica oleracea var. sabellica*) as miracle food with special reference to therapeutic and nutraceuticals perspective

**DOI:** 10.1002/fsn3.2476

**Published:** 2021-11-05

Retraction: “Kale (*Brassica oleracea var. sabellica*) as miracle food with special reference to therapeutic and nutraceuticals perspective”, by Waseem Khalid, Muhammad Sajid Arshad, Muhammad Imran, Rabia Shabir Ahmad, Ali Imran, Tahira Batool Qaisrani, Zubia Asghar, Adnan Husain, Faqir Muhammad Anjum, Hafiz Ansar Rasul Suleria, *Food Science & Nutrition*, 2022. The above article, published online on 5 November2021 in EarlyView section on Wiley Online Library (https://doi.org/10.1002/fsn3.2476), has been retracted by agreement between the authors, the journal’s Editor‐in‐Chief, Prof. Y. Martin Lo, and Wiley Periodicals, LLC. The retraction has been agreed as the editorial office found unambiguous evidence that the manuscript was accepted solely based on compromised reviewer reports.

